# Development and Validation of an Automated Magneto-Controlled Pretreatment for Chromatography-Free Detection of Aflatoxin B_1_ in Cereals and Oils through Atomic Absorption Spectroscopy

**DOI:** 10.3390/toxins14070454

**Published:** 2022-07-01

**Authors:** Jin Ye, Mengyao Zheng, Haihua Ma, Zhihong Xuan, Wei Tian, Hongmei Liu, Songxue Wang, Yuan Zhang

**Affiliations:** 1 Key Laboratory of Grain Information Processing and Control, Henan University of Technology, Ministry of Education, Zhengzhou 450001, China; yj@ags.ac.cn; 2 Henan Key Laboratory of Grain Photoelectric Detection and Control, Henan University of Technology, Zhengzhou 450001, China; 3 College of Information Science and Engineering, Henan University of Technology, Zhengzhou 450001, China; 4 Academy of National Food and Strategic Reserves Administration, Beijing 102600, China; zhengmengyao2021@126.com (M.Z.); xzh@ags.ac.cn (Z.X.); tw@ags.ac.cn (W.T.); lhm@ags.ac.cn (H.L.); wsx@ags.ac.cn (S.W.); 5 College of Food Science and Engineering, Henan University of Technology, Zhengzhou 450001, China

**Keywords:** aflatoxin B_1_, atomic absorption spectroscopy, automated pretreatment system, quantum dots, magnetic-based immunosensor

## Abstract

A chromatography-free detection of aflatoxin B_1_ (AFB_1_) in cereals and oils through atomic absorption spectroscopy (AAS) has been developed using quantum dots and immunomagnetic beads. A magneto-controlled pretreatment platform for automatic purification, labeling, and digestion was constructed, and AFB_1_ detection through AAS was enabled. Under optimal conditions, this immunoassay exhibited high sensitivity for AFB_1_ detection, with limits of detection as low as 0.04 μg/kg and a linear dynamic range of 2.5–240 μg/kg. The recoveries for four different food matrices ranged from 92.6% to 108.7%, with intra- and inter-day standard deviations of 0.7–6.3% and 0.6–6.9%, respectively. The method was successfully applied to the detection of AFB_1_ in husked rice, maize, and polished rice samples, and the detection results were not significantly different from those of liquid chromatography-tandem mass spectrometry. The proposed method realized the detection of mycotoxins through AAS for the first time. It provides a new route for AFB_1_ detection, expands the application scope of AAS, and provides a reference for the simultaneous determination of multiple poisonous compounds (such as mycotoxins and heavy metals).

## 1. Introduction

Aflatoxins (AFTs) are the most common mycotoxins [1] produced by *Aspergillus flavus*, A. *parasiticus*, and A. *nomius* [2,3,4] in cereal grains in the entire food chain, from farm to factory, under favorable temperature and humidity conditions [5]. AFTs include aflatoxin B_1_ (AFB_1_), aflatoxin B_2_, aflatoxin G_1_, and aflatoxin G_2_. Among these, AFB_1_ is the most toxic and has the highest detection frequency [6,7,8]. The AFB_1_ contamination of food products is the most common contamination problem in the food chain and is potentially hazardous to humans and animals because it causes high carcinogenicity, mutagenicity, suppression of immunity, and liver damage [8,9]. To minimize the human health risk, many countries and regions have set extremely low maximum limits (MLs) for AFB_1_ in food. For example, the European Commission established 2–12 μg/kg as the ML of AFB_1_ in food, while China established 5–20 μg/kg as the ML of AFB_1_ [10,11]. Therefore, it is necessary to address the contamination problem by assessing the risk of AFB_1_ contamination in the food chain for food safety and human health protection.

Currently, conventional analytical approaches used for AFB_1_ monitoring in food include advanced instrumental analysis and fast detection techniques. Instrumental analysis methods mainly rely on chromatography-based techniques, such as high-performance liquid chromatography [11,12,13] and liquid chromatography–tandem mass spectrometry (LC-MS/MS) [14,15,16], because of their high selectivity, excellent accuracy, and reproducibility. However, these analytical procedures have several limitations. Chromatographic methods incur high equipment costs and involve complicated and time-consuming sample operation and analysis procedures, which limit their use to skilled operators [10]. In addition, chromatography also requires consuming some chromatographic consumables, such as chromatographic columns, etc. These limitations are overcome by fast detection methods, such as thin-layer chromatography [17,18,19,20,21], colloidal gold immunochromatography [22,23,24,25], and enzyme-linked immunosorbent assay [26,27,28]; these are commonly used in the routine monitoring of AFB_1_, especially for on-site or field detection, because of their low cost and convenience of operation. However, because limited sample clean-up strategies are used, the fast detection methods are often affected by matrix effects, leading to false-positive/negative results. Hence, a rapid, accurate, sensitive, and robust methodology based on inexpensive and easy-to-operate techniques for high-frequency and precise monitoring of trace AFB_1_ must be developed. 

Atomic absorption spectroscopy (AAS) is one of the most commonly used techniques for tracing heavy-metal element determination in food, owing to its robustness, accuracy, speed, and simplicity. However, AAS-based methods have received considerably less attention in the analysis of organic contaminants such as mycotoxins primarily because they are typically regarded as “elemental analytical methods [29].” In recent years, with the continuous development of molecular labeling methods and the high specificity of antigen–antibody reactions, the target signal (organic contaminant signal) to be detected can be successfully converted to metal ion signals, which can be detected by using an elemental analysis method. Wang [30] and Hansen [31] et al. completed the conversion of DNA and protein signals to metal ion signals with the help of modified quantum dots, which allowed for the detection of target DNA and proteins by electrochemical means. This suggests that the gap between inorganic and organic analyses can be bridged. In addition, nanomaterials such as quantum dots (QDs) are aggregates of atoms and molecules on the nanometer scale, which can generate a large number of atoms after digestion, thereby improving signal amplification and detection sensitivity [32,33]. Furthermore, an AAS-based analysis system has a lower instrument cost than chromatography-based systems. As a necessary instrument for food analysis labs (many end users), the analytical potential of AAS for mycotoxins is worth exploring. 

In conventional analysis, several manual steps such as extraction, filtering, and purification are required. Consequently, it is only applicable to high-end laboratories with skilled technicians. Previously, we reported a fully automated pretreatment platform for sample enrichment, purification, and elution based on immunoaffinity magnetic beads (IMBs) [10,34,35,36], which consumes less time, costs, and labor as well as introduces fewer errors compared to manual processing of mycotoxin assays. 

In this study, we proposed an AAS-based analysis method for the specific and sensitive detection of AFB_1_ in complex food samples by using QDs as labels. To overcome the limitations of cumbersome steps, in this work, we used the IMB-based sample pretreatment platform and the AAS system in combination to generate new automated magneto-controlled analytical approaches for achieving programmable immunoassay operations, including automatic enrichment, purification, QD labeling, release, and analysis. In-house magneto-controlled systems present significant advantages over other immunosensors, which minimize labor and eliminate operational errors. The proposed method overcomes the shortcomings of existing methods and has several advantages such as simple manipulation, high sensitivity, acceptable linear calibration range and reproducibility, low-cost detection, ease of automation, and high analyte throughput. To the best of our knowledge, this study is the first to use AAS to detect AFB_1_ in different food matrices.

## 2. Results and Discussion

### 2.1. Method Principle

**Figure 1:**
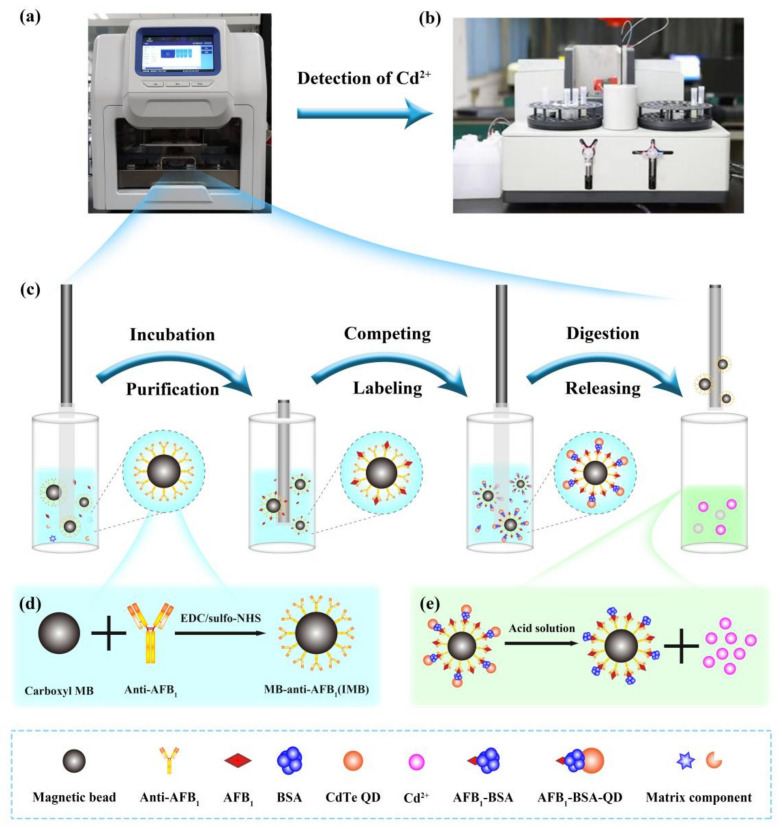
Schematic diagrams of the integrated detection platform: (**a**) automatic purifier; (**b**) atomic absorption spectrometer; (**c**) the purification process of the automatic purifier; (**d**) synthesis of immunomagnetic beads (IMB); (**e**) release of Cd^2+^ from quantum dot digestion.

In this study, by exploiting the specificity of immunomagnetic beads and the signal amplification effect of QDs, an automatic platform for enrichment, purification, labeling, and digestion was constructed to detect AFB_1_ through AAS. This principle is outlined in Figure 1. AFB_1_ in the sample extract was first specifically captured by IMBs according to the antigen–antibody reaction. After the reaction, the IMBs were transferred to wash wells to prevent nonspecific adsorption of the matrix from affecting subsequent steps and to improve the stability of the method. The complex of AFB_1_ and the IMBs was then transferred to a reaction well, where the bovine serum albumin (BSA)-assisted QD-labeled intact AFB_1_ antigen (AFB_1_-BSA-QD) is located, and AFB_1_-BSA-QD occupied the remaining adsorption sites. After washing, the IMB complexes were transferred to digestion well, and the QDs captured on the IMB were digested by the acid solution, releasing the corresponding metal ions (Cd^2+^). When the content of AFB_1_ in solution is high, a low amount of the AFB_1_-BSA-QD conjugate should be bound to the IMB, and low content of Cd^2+^ must be released via digestion. Therefore, the content of Cd^2+^ in the digestion solution is inversely proportional to the content of AFB_1_. Finally, the released Cd^2+^ was measured using an atomic absorption spectrometer to detect AFB_1_.

### 2.2. Optimization of Experimental Parameters

#### 2.2.1. Extraction Solution

**Figure 2:**
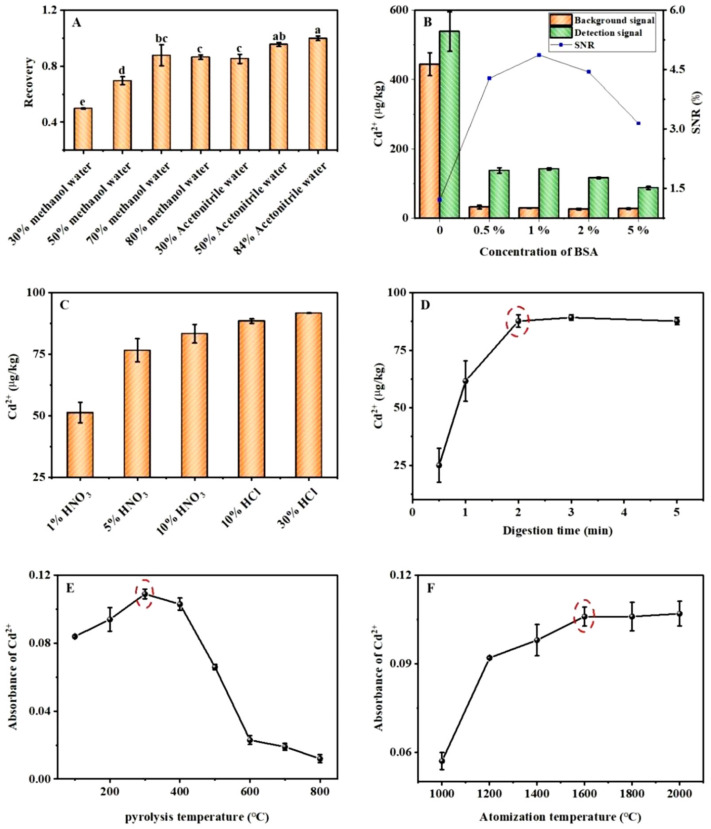
Optimization results of (**A**) extraction solution (the same small letters indicate no significant difference, *p* > 0.05), (**B**) BSA concentration, (**C**) digestion solution, (**D**) digestion time, (**E**) pyrolysis temperature, and (**F**) atomization temperature. The red circles mark the best conditions.

Adequate extraction of the target from the sample matrix is the first step toward realizing accurate detection. In this experiment, blank rice was spiked with AFB_1_ at a known concentration and different proportions of methanol and acetonitrile (30–80% methanol in water and 30–84% acetonitrile in water) were used to investigate the extraction efficiencies of AFB_1_. The detection results were compared using the least significant difference method; the results are shown in Figure 2A. When 50% and 84% acetonitrile in water were used as the extract, the recovery rate of AFB_1_ was close to 100%, and there was no significant difference between the two. To reduce the use of organic solvents, 50% acetonitrile in water was selected as the optimal extraction solution for AFB_1_.

#### 2.2.2. Nonspecific Adsorption

Nonspecific adsorption in the sample matrix leads to strong background interference. To eliminate background interference, BSA was used to block the inactive sites; however, the BSA concentration was too high, which negatively affected the strong binding. Therefore, the concentration of BSA in the reaction system was optimized; the results are shown in Figure 2B. As the BSA concentration increased from 0% to 5%, the signal-to-noise ratio of Cd^2+^ first increased and then decreased; when the BSA concentration was 1%, the signal-to-noise ratio of Cd^2+^ reached the maximum value. Therefore, 1% was determined to be the optimal BSA concentration.

#### 2.2.3. Digestion Conditions

The digestion effect of acid on the QDs directly affects the generation of Cd^2+^. To maximize the digestion of the QDs and generate Cd^2+^ in the shortest amount of time, the digestion solution and time were optimized. Figure 2C shows that as the concentration of HNO_3_ solution increased from 1% to 10%, the concentration of Cd^2+^ trended upward, whereas as the concentration of the HCl solution increased from 10% to 30%, the concentration of Cd^2+^ exhibited only a slight upward trend. Therefore, 10% HCl was determined to be the optimal digestion solution. The digestion time was optimized by using an optimal digestion solution. As shown in Figure 2D, with the extension of the digestion time, the concentration of Cd^2+^ gradually increased, reaching a maximum at 2 min, and then gradually stabilized. Therefore, 2 min was determined as the optimal digestion time.

#### 2.2.4. Atomic Absorption Conditions

Pyrolysis is a crucial stage in sample pretreatment. To ensure that the measured elements are not lost, an appropriate pyrolysis temperature at which matrix interference and pyrolysis time are reduced must be chosen. As shown in Figure 2E, with an increase in the pyrolysis temperature, the absorbance value of Cd^2+^ first increased and then decreased, reaching a maximum of 300 °C. This is because an appropriate increase in the pyrolysis temperature can allow the removal of the coexisting matrix and interfering components, the reduction or elimination of the background peak, and an increase in the absorbance value. When the temperature increased beyond a certain level, the pyrolysis-induced loss of Cd^2+^ increased, resulting in a decrease in the absorbance value. Therefore, 300 °C was determined to be the optimal pyrolysis temperature.

The atomization temperature is determined on the basis of the properties of the elements and their corresponding compounds. If the temperature is too high, the sensitivity is reduced, and the service life of the graphite tube is shortened. A low atomization temperature can prolong the service life of the graphite tube. However, if the atomization temperature is too low, the complete atomization of the measured element cannot be guaranteed, resulting in decreased sensitivity and low reproducibility. As shown in Figure 2F, with an increase in the atomization temperature, the absorbance of Cd^2+^ gradually increased, reached a maximum at 1600 °C, and then gradually became stable. Therefore, 1600 °C was determined to be the optimal atomization temperature.

### 2.3. Establishment and Specificity of Standard Curves in a Standard Solution and Four Types of Matrix Fluids

**Figure 3:**
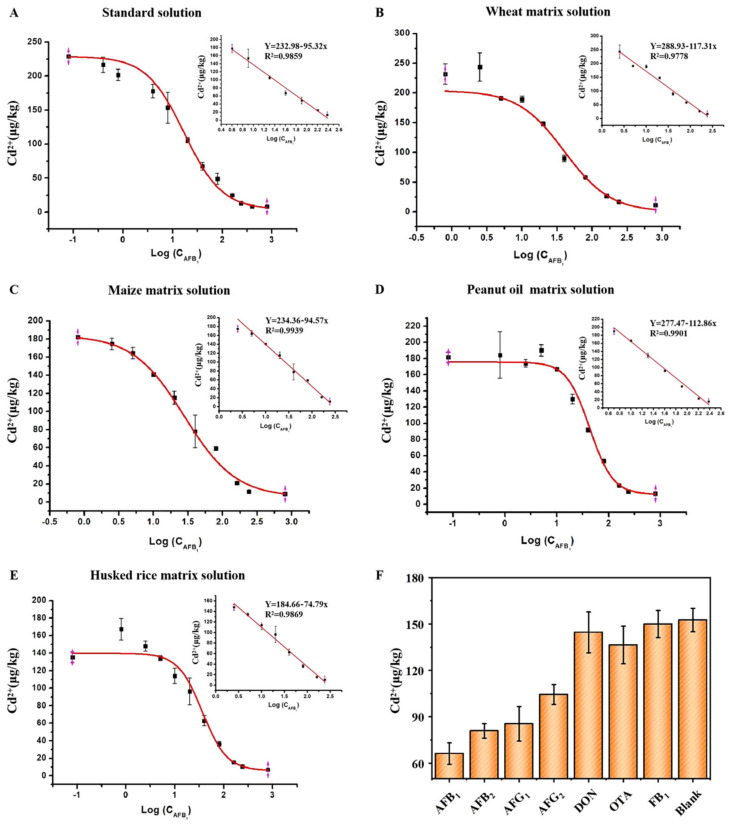
S-curves and standard curves of (**A**) a standard solution and (**B**–**E**) four matrix solutions spiked with aflatoxin B_1_ (AFB_1_). (**F**) Specificity detection results.

Under optimal conditions, taking the log value of the AFB_1_ concentration as the abscissa and the Cd^2+^ concentration as the ordinate, the S-shaped curve and standard curve of the spiked standard solution and the four matrix solutions can be obtained. The results for the spiked standard solutions are shown in Figure 3A. The correlation coefficient of the fitted curve was 0.9986, and when the concentration of AFB_1_ was 5–240 μg/kg, the log value of its concentration was linearly related to the concentration of Cd^2+^ with an R^2^ of 0.9859. The limits of detection and quantification were 0.04 and 0.12 μg/kg, respectively. The standard addition results of the sample matrix (wheat, corn, peanut oil, and husked rice) are shown in Figure 3B–E, and the correlation coefficients of the curve fits were all greater than 0.99, approximately 1. In addition, the log value of the AFB_1_ concentration was linearly related to the concentration of Cd^2+^ within a certain range. The relevant parameters are listed in Table 1.

To evaluate the selectivity of the detection platform for AFB_1_, three analogs of AFB_1,_ including aflatoxin B_2_ (AFB_2_), aflatoxin G_1_ (AFG_1_), and aflatoxin G_2_ (AFG_2_), and common mycotoxins deoxynivalenol (DON) ochratoxin (OTA)and fumonisin B_1_ (FB_1_) were investigated. All toxin concentrations were 20 μg/kg. Figure 3F shows that AFB_1_ produced a significantly lower metal ion signal than that of the blank control, while the signal variations induced by the DON, OTA, and FB_1_ could be ignored. Although the metal ion signal values of the three analogs similar in structure to AFB_1_ are slightly lower, in the future, the use of antibodies with better specificity can reduce the cross-reactivity to AFB_1_ structural analogs and improve the specificity of the method.

### 2.4. Accuracy, Repeatability, and Reproducibility of the AAS Method

To evaluate the accuracy, repeatability, and reproducibility of the AAS method, four samples of corn, wheat, husked rice, and peanut oil were used, and they were spiked with three concentration gradients of low, medium, and high matrices (10, 20, and 40 μg/kg for maize and peanut oil; 2.5, 5, and 10 μg/kg for wheat; and 5, 10, and 20 μg/kg for husked rice). The recovery rates and intra- and inter-day relative standard deviations were measured and calculated; the results are shown in Table 2. The recoveries for the four matrices ranged from 92.6% to 108.7%, with intra- and inter-day standard deviations of 0.7–6.3% and 0.6–6.9%, respectively. These data demonstrate that the proposed method has high accuracy and precision.

The accuracy of this method was also verified using (certified) reference material. In this study, three AFB_1_ reference materials (maize, peanut oil, and husked rice) provided by ASAG were selected. Among them, the uncertainty range of maize (GBW(E)100386) is 24–30 μg/kg; the uncertainty range of peanut oil(JTZK-002) is 13.9–17.7 μg/kg; the uncertainty range of husked rice(JTZK-007) is 22.1–29.9 μg/kg. It can be seen from Table 3 that all the detected values of the AFB_1_ standard material are within the uncertainty range.

### 2.5. Analysis of Real Samples

To verify the feasibility of the proposed method, naturally contaminated husked rice, maize, and rice samples were analyzed, and AFB_1_ concentrations were determined by the proposed method and LC-MS/MS [37]. A paired sample T-test was used to compare whether there were significant differences in the detection results of the two methods. The measurement and analysis results are summarized in Table 4. The Sig. (two-tailed) values of the husked rice, maize, and rice samples are all greater than 0.05. There is no significant difference in the detection results of AFB_1_ between this method and LC-MS/MS; thus, the proposed method can be used for the detection of AFB_1_ in actual samples.

## 3. Conclusions

In this study, an automatic platform for enrichment, purification, labeling, and digestion was successfully constructed, allowing for the detection of AFB_1_ by AAS. The experimental data showed that the method has high sensitivity, specificity, accuracy, and precision, a wide range of linearity, and is applicable for the analysis of most samples. There was no significant difference in the detection results of the proposed method and LC-MS/MS during real sample analysis, indicating that this method can be used for the detection of AFB_1_ in food. An automatic magnetron pretreatment system based on a quantum dot immunosensor was combined with an atomic absorption spectrometer to realize signal conversion between mycotoxin AFB_1_ and metal ion Cd^2+^. This method expands the application scope of AAS and provides a reference for the simultaneous determination of multiple poisonous compounds (such as mycotoxins and heavy metals).

## 4. Materials and Methods

### 4.1. Materials

AFB_1_, dimethyl formamide (DMF), pyridine,O-(carboxymethyl) hydroxylamine hemihydrochloride (CMO), trichloromethane, 1-ethyl-3-(3-dimethylaminopropyl) carbodiimide hydrochloride (EDC), sodium hydroxide, N-hydroxysuccinimide (NHS), hydrochloric acid (HCl), nitric acid (HNO_3_), bovine serum albumin (BSA), and phosphate buffer (PBS) were purchased from SigmaAldrich (St. Louis, MO, USA). HPLC grade methanol (MeOH) was obtained from Merck (Darmstadt, Germany). Deionized water (H_2_O) was purchased from Watsons (Hong Kong, China). Blank maize, rice, wheat, husked rice, and peanut oil samples were obtained from a market in China. Certified reference material and reference materials are provided by the Academy of National Food and Strategic Reserves Administration(ASAG) (Beijing, China; GBW(E)100386, JTZK-007, JTZK-002).

### 4.2. Synthesis of Immunomagnetic Beads (IMB)

First, take 500 μL of NHS-activated carboxyl magnetic beads, discard the supernatant after magnetic separation, add 1 mL of dilute hydrochloric acid pre-cooled at 2–8 °C and mix well. After magnetic separation, the supernatant was discarded, and 500 μL of 2 mg/mL AFB_1_ antibody was added. After homogeneous, it was placed on a mixer for 2 h at room temperature. Next, the reaction mixture was magnetically separated, the supernatant was discarded, 1 mL of blocking buffer (Tris-HCl) was added and mixed, and then placed on a mixer for 2 h at room temperature to form IMB. Finally, the mixture was magnetically separated, and the supernatant was discarded, washed with 1 mL PBST (2 times) and 1 mL PBS (1 time) IMB, and then added 500 μL PBS to resuspend and stored at 4℃ for later use.

### 4.3. Synthesis of Aflatoxin Haptens (AFB_1_-CMO)

Since there is no active group on the surface of the AFB_1_ molecule that can be coupled with the protein, the AFB_1_ molecule needs to be derivatized before coupling with BSA. We used O-(carboxymethyl)hydroxylamine hemihydrochloride (CMO) as a derivatizing agent to make AFB_1_ carry a carboxyl group, which can then react with the amino group on BSA to achieve the coupling effect. The derivatization scheme was as follows: 1 mg of AFB_1_ was dissolved in 0.6 mL of methanol–water–pyridine (4:1:1) solution, 2 mg of CMO was added, and the reaction was carried out under magnetic stirring in a water bath at 70 °C for 6 h and allowed to stand overnight at room temperature in the dark. The reaction solution was blown dry with nitrogen, and the precipitate was dissolved in 1 mL of chloroform solution and extracted three times with an equal volume of ultrapure water. The organic phase was collected and dried with nitrogen, and the precipitate was dissolved in 200 μL of Dimethyl Formamide (DMF) solution, which was the derivative product.

### 4.4. Synthesis of Aflatoxin Complete Antigen (AFB_1_-BSA)

The complete antigen synthesis is based on the hapten synthesis by adding the coupling protein BSA. The coupled protein BSA was first dissolved in carbonate buffer. Then, 1 mg of AFB_1_-CMO was dissolved in 200 μL of DMF solution, 2.4 mg of EDC and 1.3 mg of NHS were added, and the reaction was conducted under magnetic stirring at room temperature for 12 h. The reaction solution was added dropwise to the carrier protein solution (BSA: 7.54 mg), and the reaction was magnetically stirred at room temperature overnight. The coupled product was ultrafiltered with an ultrafiltration tube, and the retentate was resuspended in PBS and stored at −20 °C for later use. The successful synthesis of AFB_1_-BSA was characterized by the UV-Vis absorption spectra of BSA and AFB_1_-BSA. The characteristic absorption peak of BSA is at 278 nm, the characteristic absorption peak of AFB_1_ is at 265 nm and 360 nm, and the characteristic absorption peak of AFB_1_-BSA is between 265–278 nm, which is mainly due to the superposition of the characteristic absorption peaks of AFB_1_ and BSA. The successful synthesis of the complete antigen can be well characterized by the UV-Vis absorption pattern.

### 4.5. Coupling of Pegylated Quantum Dots with Complete Antigen AFB_1_-BSA

The cross-linking principle of polyethylene glycol-modified quantum dots and AFB_1_-BSA is based on the fact that after heat treatment of BSA, a part of the internal hydrophobic structure will be exposed, which can be adsorbed with PEG on the surface of quantum dots. The cross-linking steps are as follows: 22.2 µL of 100 mM PBS was added to 200 µL of 1 µM PEG-modified quantum dot solution to prepare a 10 mM PBS quantum dot solution (pH 7.4). Subsequently, 67 µg of AFB_1_-BSA was added and mixed, followed by a boiling water bath for 10 min, centrifugation at 25,000 rpm for 15 min, the supernatant was discarded, and the quantum dots were resuspended.

### 4.6. Sample Automatic Processing

Representative samples were thoroughly ground and homogenized according to the Codex General Guidelines on Sampling from the FAO and WHO with minor modifications [38].Cereal samples are treated as follows: 3 points are randomly selected for sampling, and the sampling amount of each point is 0.5 kg as the laboratory sample size. All laboratory samples were pulverized with a particle size of 0.5 mm. After fully mixing, 5 g of the sample was weighed for processing. Oil samples are treated as follows: after the peanut oil sample is fully mixed, 5 g of the sample is weighed for processing. Sequentially, 5 g [37] sample and 20 mL extraction solution were vortexed at 2500 rpm for 20 min in a centrifuge tube (50-mL). Finally, the centrifuge tube was centrifuged at 7000 rpm for 5 min, with the supernatants for further analysis. Proper mixing frequency, mixing amplitude, and sufficient reaction time can ensure sufficient reaction, washing, and elution of the sample. In order to prevent the solvent from splashing out during the mixing process, the mixing range was set to 80%, and other reaction conditions were shown in Table 5. After the pretreatment, the eluates in 5 wells were collected for detection by atomic absorption spectrometer or LC-MS/MS [37].

### 4.7. Atomic Absorption Detection

The determination of cadmium (Cd^2+^) was done by using the graphite furnace atomic absorption spectrometer (CPG2S, China). A Cd hollow cathode lamp operating at the 228.8 nm analytical line (4 mA current and a 0.8 nm spectral bandpass) was used for absorbance measurement, and the deuterium lamp was used to correct the background. Other experimental conditions and heating procedures are shown in Table 6.

### 4.8. Method Verification

The linear range, limit of detection (LOD), limit of quantification (LOQ), recovery, intra-day relative standard deviation, and inter-day relative standard deviation were determined for the method, and real samples were analyzed together by LC-MS/MS. Where the limit of detection (3 σ/s) and the limit of quantification (10 σ/s) are calculated from the calibration, “σ” is the standard deviation of the 11 blank measurements, and “s” is the slope of the calibration curve.

## Figures and Tables

**Table 1:** Analysis parameters related to the sigmoid curves and standard curves of the standard solution and the four matrix solutions.

	Curve Range (μg/kg)	Curve Coefficient	Linear Range (μg/kg)	Linear Coefficient
Standard solution	0.08–800	0.9986	5–240	0.9859
Wheat	0.8–800	0.9944	2.5–240	0.9778
Maize	0.8–800	0.9951	2.5–240	0.9939
Peanut oil	0.08–800	0.9977	2.5–240	0.9901
Husked rice	0.08–800	0.9989	5–240	0.9869

**Table 2:** Recoveries and intra- and inter-day relative standard deviations of the sample matrices.

Matrix	Recovery ± RSD (%, *n* = 3)			Inter-Day RSD (%)
	Low	Medium	High	
Maize	99.5 ± 4.2	94.9 ± 3.2	101.7 ± 1.7	6.2
Wheat	92.9 ± 4.7	103.8 ± 2	100.3 ± 4.4	5.8
Husked rice	94.7 ± 5.2	95.8 ± 0.8	92.6 ± 4.8	0.6
Peanut oil	108.7 ± 6.3	104.4 ± 1.8	101.9 ± 0.7	6.9

**Table 3:** The detection results for the certified reference material and reference materials (*n* = 3).

AFB_1_ Reference Material	Lot Number	Detected Amount (µg/kg)	Certificate Value (µg/kg) ± SD
Maize	GBW(E)100386	26.5	27 ± 3
Peanut oil	JTZK-007	15.2	15.8 ± 1.9
Husked rice	JTZK-002	25.5	26 ± 3.9

**Table 4:** Analyses of the results of the AAS and liquid chromatography–tandem mass spectrometry (LC-MS/MS) methods.

Sample	This Method (µg/kg)	LC-MS/MS (µg/kg)	t	df	Sig. (Two-Tailed)
Husked rice	31.70	31.88	0.305	2	0.789
	32.31	32.40			
	33.77	33.32			
Maize	38.32	31.00	1.855	2	0.205
	31.75	29.00			
	31.75	31.00			
Rice	6.90	8.60	1.095	2	0.388
	16.69	11.30			
	14.59	11.40			

**Table 5:** The mixing frequency and sequence of the automatic clean-up procedure.

Step	Well	Mixing Time/min	Collection Time/min	Mixing Frequency /Hz
Transfer	2	1.0	0.5	6.5
Reaction	1	3.0	1.0	1.5
Wash 1	2	1.0	0.5	6.5
Competing	3	1.0	0.5	6.5
Wash 2	4	1.0	0.5	6.5
Digestion	5	1.0	0.5	7.5
Collection	2	1.0	0.5	6.5

**Table 6:** Instrumental operating conditions and heating program for the determination of Cd^2+^.

Spectrometer Conditions		Heating Program				
	Cd	Step	Temperature (°C)	Ramp (s)	Hold (s)	Argon
Wavelength (nm)	228.8	Drying 1	75	5	2	ON
Bandpass (nm)	0.8	Drying 2	90	5	2	ON
sample volume (μL)	12	Drying 3	110	10	2	ON
Lamp current (mA)	4	Pyrolysis	300	5	5	ON
		Atomization	1600	2	1	OFF
		Cleaning	1650	1	1	ON

## Data Availability

Not applicable.
